# A Special Issue for the 2nd International Biological Mass Spectrometry (BMS) Symposium 2018 in Kyoto

**DOI:** 10.5702/massspectrometry.K0012

**Published:** 2020-02-05

**Authors:** Chen Lee Chuin, Tohru Yamagaki

**Affiliations:** 1 University of Yamanashi; 2 Chair of the Organizing Committees; 3 Suntory Foundation for Life Sciences, Bioorganic Research Institute

We have the great pleasure to present this special issue consisting of selected peer-reviewed articles that were presented at the 2nd international biological mass spectrometry symposium held from October 26th to 27th, 2018 at the Shimadzu HQ in Kyoto. The symposium was organized by the biological mass spectrometry (BMS) division of the Mass Spectrometry Society of Japan. The focus of the symposium was on “Understanding Life by MS,” and featured four invited keynote lectures delivered by leading experts from different countries and eight topics by local researchers. There were over 80 attendees at the symposium representing various disciplines who engaged in vigorous discussions and interactions. The topics delivered at the symposium are listed as follows:

Keynote lectures:
  ·Cristian Arsene (Physikalisch-Technische Bundesanstalt Braunschweig und Berlin, Germany)
    Development of a reference method for the determination of HbA2.
  ·Joanne Yew (University of Hawaii, USA)
    Deciphering the lipid language of insects with ambient mass spectrometry.
  ·Peter Nemes (University of Maryland, MD, USA)
    Capillary electrophoresis for single-cell mass spectrometry of the early developing embryo.
  ·Brendan MacLean (University of Washington, WA, USA)
    On the building of the Skyline targeted mass specrometry software.
Regular oral sessions:
  ·Tomoya Kinumi (AIST, Japan)
    Standard materials and measurement methods leading to precise peptide and protein measurements.
  ·Tadayuki Ogawa (Univ. Tokyo, Japan)
    A smart nano-machine that demolishes microtubule cytoskeletons in cells.
  ·Naoyuki Sugiyama (Kyoto Univ., Japan)
    Phosphoproteome and kinome profiling using nanoLC-MS/MS.
  ·Lee Chuin Chen (Univ. Yamanashi, Japan)
    Hyphenation of high-temperature capillary liquid chromatography with electrospray ionization mass spectrometry: Technical challenges & future prospects.
  ·Hidenori Takahashi (Shimadzu Co., Japan)
    Structural analysis of biomolecules using Hydrogen Attachment/abstraction Dissociation (HAD) and related novel radical induced dissociation techniques.
  ·Fumio Matsuda (Osaka Univ., Japan)
    ^13^C-Metabolic flux analysis of the metabolism of cancer cells.
  ·Susumu Uchiyama (Osaka Univ., Japan)
    Roles of mass spectrometry in protein science and engineering.
  ·Kentaro Ishii (Nat. Institutes of Natural Sciences, Japan)
    Native MS: mass spectrometry for characterizing non-covalent molecular complexs.

In addition to the regular oral session, seventeen students and young researchers also took part in short 5-min oral presentations and poster sessions. One symposium research award and three student symposium awards were presented for outstanding presentations during the symposium. The award evaluation committee consisted of nine board members of the BMS division. The winners were announced at the award ceremony which was held during the banquet.

The winner of the Symposium Research Award is:
  ·Kanae Teramoto (Shimadzu Co., Kyoto)
    MALDI-MS proteotyping of *Cutibacterium acnes*.
The winners of the Student Symposium Award are:
  ·Norhiedayah Rosli (Univ. Yamanashi)
    Development of high-temperature liquid chromatography ESI-MS.
  ·Haruhiko Maruyama (Osaka Univ.)
    Analysis of metabolic responses to paclitaxel treatment in central carbon metabolism of cancer cells.
  ·Hiroaki Oyama (Osaka Univ.)
    Stability and structural changes of an antibody in different buffers.

Finally, we wish to take this opportunity to thank all of the members of the organizing committee: Susumu Uchiyama (Osaka Univ.), Tomoya Kinumi (AIST), Fumio Matsuda (Osaka Univ.), Rika Miyake (Osaka Univ.), Mayuko Morita (Kyoto Pref. Univ. of Medicine), and all of the reviewers for their work. It is our hope that this special issue will be useful as reference materials for mass spectrometrists and life science researchers.

**Figure 1:**
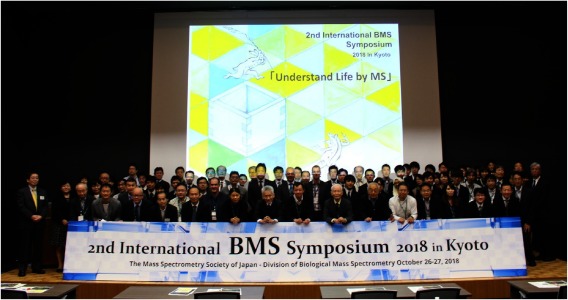
Fig. 1. Attendees of the 2nd International Biological Mass Spectrometry (BMS) Symposium 2018 in Kyoto.

